# Organoids for the Study of Retinal Development and Developmental Abnormalities

**DOI:** 10.3389/fncel.2021.667880

**Published:** 2021-05-05

**Authors:** Anne Vielle, Yuna K. Park, Conner Secora, M. Natalia Vergara

**Affiliations:** ^1^CellSight Ocular Stem Cell and Regeneration Program, Sue Anschutz-Rodgers Eye Center, University of Colorado School of Medicine, Aurora, CO, United States; ^2^Linda Crnic Institute for Down Syndrome, Aurora, CO, United States; ^3^Master of Science in Modern Human Anatomy Program, Aurora, CO, United States

**Keywords:** retina, development, stem cells, organoids, congenital abnormalities

## Abstract

The cumulative knowledge of retina development has been instrumental in the generation of retinal organoid systems from pluripotent stem cells; and these three-dimensional organoid models, in turn, have provided unprecedented opportunities for retinal research and translational applications, including the ability to model disease in a human setting and to apply these models to the development and validation of therapeutic drugs. In this review article, we examine how retinal organoids can also contribute to our understanding of retinal developmental mechanisms, how this knowledge can be applied to modeling developmental abnormalities, and highlight some of the avenues that remain to be explored.

## Introduction

**Figure 1:**
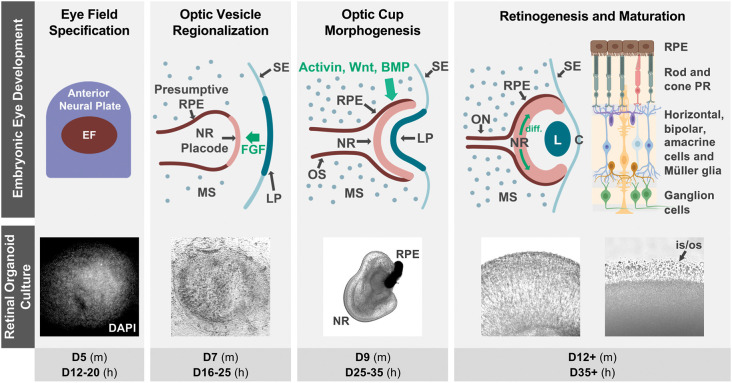
Retinal organoid cultures recapitulate *in vivo* retinal development. The top row illustrates some of the hallmarks of retinal development *in vivo*, as well as some key signaling interactions that specify the neural retina and RPE territories in the optic vesicle (notice that interactions contributing to ventral optic vesicle/optic stalk specification are not described, as they are outside of the scope of this review). Fluorescence (DAPI) and bright field micrographs in the middle row show examples of retinal organoid morphology at each of the corresponding developmental stages (retinal organoids were derived from hiPSC using the Zhong et al., 2014 protocol). The bottom row indicates the approximate timing of each developmental step in mouse (m) and human (h) retinal organoid cultures. The retinal diagram was generated using BioRender. Abbreviations: BMP, bone morphogenetic protein; C, cornea; D, days of differentiation; diff., differentiation wave; EF, eye field; FGF, fibroblast growth factor; L, lens; LP, lens placode; MS, mesenchyme; NR, neural retina; ON, optic nerve; OS, optic stalk; is/os, photoreceptor inner and outer segments; PR, photoreceptors; RPE, retinal pigmented epithelium; SE, surface ectoderm.

The vertebrate retina is an extension of the central nervous system composed of seven main types of neurons and glia specialized for visual function. Its delicate and complex organization arises during embryonic development through tightly spatiotemporally regulated mechanisms that are highly conserved among vertebrates (Figure 1).

The retina originates from the ventral diencephalon, where a group of cells begins to co-express a set of transcription factors including Pax6, Rax, Six3, Six6, and Lhx2, and becomes specified as the eye field (Zuber et al., 2003; Byerly and Blackshaw, 2009). This eye field evaginates bilaterally to form the optic vesicles, which grow distally towards the surface ectoderm where inductive signals from the lens placode contribute to the specification of the retinal placode. Next is a concerted invagination of both tissues to form the lens vesicle and the bilayered optic cup (Figure 1). As the inner layer continues to proliferate and become established as the retinal neuroepithelium, interactions with the extraocular mesenchyme and surface ectoderm specify retinal pigmented epithelial (RPE) fate in the outer layer of the optic cup (Adler and Canto-Soler, 2007; Fuhrmann, 2010; Heavner and Pevny, 2012).

Retinogenesis begins at the posterior pole of the retinal neuroepithelium, spreading anteriorly as a wave, with cell cycle exit and fate specification following a sequential yet overlapping pattern that is highly conserved in vertebrates (Cepko et al., 1996). The first cells to differentiate are retinal ganglion cells (RGC), followed by cone photoreceptor precursors, amacrine and horizontal cells, and later by rod photoreceptor precursors, bipolar cells, and Müller glia (Brzezinski and Reh, 2015; Hoshino et al., 2017). Differentiation of cell subtypes and maturation follow, and synaptic formation then leads to the development of plexiform layers, completing the retinal circuitry.

Considering its complexity, it is extraordinary that this process could be reproduced *in vitro*, leading to the generation of three-dimensional (3D) retinas or retinal “organoids” from embryonic and induced pluripotent stem cells (ESC and iPSC) from different species. But how does this happen? What can we learn about retinal development by studying organoids? And can we harness the potential of organoids to gain a better understanding of congenital retinal abnormalities? Rather than compiling a comprehensive account of the literature on these topics, we highlight some key studies that illustrate the contributions of retinal organoids to answering these critical questions and propose avenues for further exploration.

## Characteristics of Retinal Organoid Models

Retinal organoids mimic the cellular composition and histoarchitecture of the native retina, including the differentiation of all major cell types organized in the characteristic trilaminar structure. Moreover, they are capable of achieving an advanced level of maturation, including the ability to respond to light stimulation and form functional synapses (Zhong et al., 2014; Wahlin et al., 2017; Hallam et al., 2018; Cowan et al., 2020). Remarkably, to date, the only way to achieve this high level of complexity is by harnessing the stem cells’ ability to recapitulate development (Figure 1; Meyer et al., 2009; Eiraku et al., 2011; Nakano et al., 2012; Zhong et al., 2014; Volkner et al., 2016; O’hara-Wright and Gonzalez-Cordero, 2020).

During retinal organoid generation, stem cell aggregates are cultured in conditions favoring their differentiation into neuroectodermal lineages. At this point, structures expressing a complete component of eye field transcription factors begin to form spontaneously (Zhong et al., 2014). These eye fields later differentiate into a central region expressing VSX2, a marker of neural retinal fate, surrounded by microphthalmia transcription factor (MITF)-positive cells representative of RPE fates, which in turn are surrounded by neural rosettes that molecularly resemble neural progenitors from the anterior neural tube, a topological arrangement that mimics the optic vesicle in early development (Figure 1; Eiraku et al., 2011; Zhong et al., 2014; Takata et al., 2017).

When these optic vesicle analogs are dissected and cultured in suspension, they fold into structures that mimic the inner layer of the optic cup. These early retinal organoids consist of a pseudostratified neuroepithelium where cell division occurs in the external (“apical”) surface, with interkinetic nuclear migration displacing nuclei radially to their final location (Eiraku et al., 2011; Zhong et al., 2014). The same pattern of apical cell division is observed even in protocols in which retinal organoid-like tissues are grown in attachment conditions. Intriguingly, those adherent retinal cultures express LGR5, a Wnt signaling activating receptor in the apical side (Singh et al., 2015). Retinogenesis in organoids also follows the sequence observed *in vivo*, including the posterior to anterior differentiation wave (Zhong et al., 2014; Vergara et al., 2017; Fligor et al., 2018; Langer et al., 2018; Luo et al., 2019). Strikingly, each of the main developmental hallmarks in retinal organoid generation roughly matches the timing of their *in vivo* counterparts in a species-specific temporal sequence (Figure 1), as confirmed by molecular, immunohistochemical, and more recently transcriptomic and single cell RNA-sequencing studies (Brooks et al., 2019; Collin et al., 2019a, b; Kaya et al., 2019; Cowan et al., 2020). However, despite the substantial conservation in cellular composition at each developmental stage, significant variability has been noted in the efficiency of retina induction among different pluripotent stem cell lines (Mellough et al., 2019; Cowan et al., 2020).

## Limitations of The Model

These characteristics make retinal organoids an attractive model to study development, especially in humans, where knowledge is sparse due to the obvious inability to perform experimental manipulations. However, there are important differences between these *in vitro* systems and the *in vivo* scenario (Aasen and Vergara, 2020). Most notably, even though RPE is formed in retinal organoid cultures, it is not juxtaposed to the apical side of the neural retina (Eiraku et al., 2011; Zhong et al., 2014; Takata et al., 2017; Singh et al., 2019). Instead, when the presumptive optic vesicle is excised during organoid generation, the RPE tissue, which remains continuous with the neural retina, lacks the attachment to the neural tube that would hold its position in the embryo* in vivo* and thus folds onto itself forming a clump at the “anterior” end of the organoid.

Retinal organoids also lack microglia, yolk-sac derivatives that invade the optic cup during the period of retinogenesis *in vivo*, and blood vessels, which are of mesodermal origin and enter the developing optic cup through the optic fissure. Organoid cultures instead favor neuroectodermal derivatives, and optic vesicle-like structures are mechanically isolated, thus preventing the formation of an optic stalk and folding of the optic fissure. Moreover, the optic nerve does not form, forcing RGC axons to remain within the organoid cavity. Eventually, as organoids continue to mature, RGCs are lost (Kaya et al., 2019; Cowan et al., 2020).

Finally, even though recent protocols have achieved an increased ratio of the cone to rod photoreceptors (Kim et al., 2019), no macula formation has yet been demonstrated in human retinal organoids, and the ability of these *in vitro* models to respond to light stimulation, albeit remarkable, is significantly smaller in magnitude than that of a mature retina (Zhong et al., 2014; Hallam et al., 2018; Cowan et al., 2020). This may be reflective of an embryonic scenario, even though studies comparing the organoid light response with the onset of function *in vivo* have not been performed.

Considering these and other deficiencies, what insights can stem cell-derived organoid systems contribute to our knowledge of the mechanisms of retinal development?

## Retinal Organoids Offer New Perspectives on Developmental Mechanisms

One of the most underappreciated lessons from organoid systems is the largely tissue-autonomous nature of their development. Decades of research have identified the mechanisms that drive retina development, yet organoid systems now bring to light new interpretations and nuances, contributing to a richer understanding of this process.

One example of this is the optic vesicle regionalization. It has been established in animal models that as the optic vesicle evaginates, fibroblast growth factor (FGF) signaling from the surface ectoderm is necessary to specify the presumptive neural retina territory by upregulating the transcription factor Vsx2, which is involved in a cross-repressive loop with MITF. In mouse and chick, removal of the surface ectoderm at the optic vesicle stage results in failure to specify a neural retina, leading to the development of microphthalmic pigmented vesicles, which can be rescued by exogenous FGF administration (Pittack et al., 1997; Hyer et al., 1998; Nguyen and Arnheiter, 2000; Horsford et al., 2005). Conversely, RPE specification and maintenance are driven by signaling molecules including Wnt, bone morphogenetic proteins, and the transforming growth factor-β (TGF-β) family member activin (Fuhrmann, 2008; Westenskow et al., 2009; Heavner and Pevny, 2012; Steinfeld et al., 2013). Studies in chicks indicate that these inductive signals originate from the extraocular mesenchyme (Fuhrmann et al., 2000; Kagiyama et al., 2005; Fuhrmann, 2010), whereas mouse studies implicate the surface ectoderm in this process (Carpenter et al., 2015).

In this context, the fact that retinal organoid development involves the consistent generation of optic vesicle-like structures composed of a central presumptive neural retina and surrounding RPE is remarkable, considering the lack of both surface ectoderm and extraocular mesenchyme. Additionally, even if these cells were produced elsewhere in the culture, the secreted factors that mediate signaling between these tissues *in vivo* would be available to the whole culture and thus would be unlikely to specify positional information. Moreover, the neural retina and RPE are known to remain plastic for some time after specification, and disruption of these external signals causes transdetermination between these two fates *in vivo*. Yet during organoid generation, once the neural retina and RPE are specified, these fates are maintained and the cells continue to differentiate and mature accordingly. This suggests that once the eye field is specified, the cells have the intrinsic ability to undergo differentiation in a manner that maintains the topological organization and subsequent maturation of the neural retina and RPE in the absence of inductive interactions with other tissues. Rather than refuting the importance of tissue interactions in optic vesicle regionalization, this seemingly contradictory finding may favor the hypothesis that interactions between embryonic tissues *in vivo* orchestrate a dynamic balance of inductive and repressive forces that restrict the endogenous differentiation program of the optic vesicle to specific spatial territories, thus ensuring the correct location and size of the developing eye structures.

Another area where retinal organoids are contributing to our understanding of eye development is in optic cup morphogenesis. Organoid systems have strengthened a model in which the morphogenetic events that result in optic vesicle to optic cup transition can be driven by intrinsic forces in the absence of external structures. Eiraku et al. (2011) used atomic force microscopy on mouse retinal organoids and identified a difference in stiffness between the developing neural retina and RPE. They proposed that RPE stiffness, coupled with the apical constriction that occurs at the hinge region between the neural retina and RPE due to the action of contractile myosin, are sufficient to drive invagination of the neural retina and shape the optic cup (Eiraku et al., 2011). This hypothesis was later refined by Carpenter et al. (2015) who, taking these findings back to an *in vivo* mouse model, proposed that proliferation of RPE cells near the hinge region driven by Wnt signaling from the surface ectoderm lengthens the stiffer RPE tissue, thus ensuring the correct curvature and shape of the optic cup (Carpenter et al., 2015).

Furthermore, retinal organoids have been used to elucidate the mechanisms that generate the mosaic of cone photoreceptor subtypes in the human retina. Eldred et al. (2018) identified thyroid hormone signaling as a regulator of the temporal switch in cone subtype specification in human retinal organoids. Moreover, they discovered that retinal organoids express thyroid hormone modulators in a temporally dynamic manner that allows them to endogenously regulate the production and ratios of S and L/M cone photoreceptors.

These are only some examples of the power of organoids to contribute to our understanding of retinal development, an exploration that is only in its beginnings.

## Potential of Organoids for Modeling Congenital Retinal Defects

Retinal organoids can also contribute to our understanding of how the disruption of developmental mechanisms leads to congenital retinal abnormalities, an approach that has already yielded promising results. Human retinal organoids generated from a patient with microphthalmia due to an R200Q mutation in VSX2 were used to investigate how this mutation leads to the pathological phenotype (Phillips et al., 2014). The study found a significant growth deficit in (R200Q)VSX2 retinal organoids compared to controls, resulting at least in part from reduced neural progenitor cell proliferation. This was accompanied by increased production of RPE at the expense of the neural retina, confirming the important pro-neural role of this gene in human retina development. Moreover, bipolar cell production and photoreceptor maturation were also compromised in mutant organoids, and RNASeq analysis identified some of the signaling pathways that seem to mediate the action of VSX2 in neural retina specification/maintenance. Numerous WNT receptors and downstream effectors, as well as TGF-β family members, were upregulated in VSX2 mutant organoids, while there was a downregulation in the pro-neurogenic FGF3, 9, and 19 genes, exemplifying how these organoids can provide insights into the mechanisms of congenital retinal abnormalities.

Additionally, retinal organoids are already contributing to our understanding of congenital glaucoma. Ohlemacher et al. (2016) compared retinal organoids derived from a patient with an E50K mutation in the Optineurin (*OPTN*) gene that causes familial forms of glaucoma, with organoids derived from control subjects, and found that RGCs in OPTN mutant organoids displayed a significant increase in caspase-3 activation. Further, treatment of OPTN RGCs with BDNF or PEDF caused a reduction in caspase-3 activation, highlighting the utility of this model as a tool for pharmacological development (Ohlemacher et al., 2016). In line with this, a later study using CRISPR/Cas9 gene editing to introduce the same OPTN(E50K) mutation in hiPSC, compared retinal organoids derived from these lines with isogenic controls. Organoids were then dissociated to further evaluate the physiological characteristics of RGCs. The results showed that RGCs differentiated from OPTN(E50K) hiPSC exhibited neurodegenerative deficits including neurite retraction, autophagy dysfunction, and increased excitability (VanderWall et al., 2020).

Retinal organoids have also been recently used to investigate how a human mutation in the *NRL* gene affects cone photoreceptor specification (Kallman et al., 2020). Mutations in this gene can cause enhanced S-cone syndrome, characterized by increased S-cone numbers at the expense of rod photoreceptors. The phenotypic manifestations range from night blindness to visual defects comparable to retinitis pigmentosa (Nishiguchi et al., 2004; Littink et al., 2018). Kallman et al. (2020) found that patient-derived retinal organoids lacking NRL are enriched in S-opsin expressing photoreceptors, and identified MEF2C as a candidate regulator of cone cell fate specification in the human retina, a function that differs from its proposed role in mouse (Kallman et al., 2020).

Furthermore, the fact that retinal organoids are capable of forming inner and outer segments, albeit immature, even in the absence of RPE juxtaposition is remarkable, and allows the possibility to study ciliopathies that affect photoreceptors leading to vision loss, such as Leber congenital amaurosis caused by mutations in the *CEP290* gene (Rachel et al., 2015; Shimada et al., 2017). For example, Parfitt et al. (2016) generated retinal organoids from patient-derived hiPSC harboring a mutation in *CEP290* and found that this mutation led to defective ciliogenesis in photoreceptors, which could be restored by antisense morpholino treatment (Parfitt et al., 2016). Additional examples of inherited retinal dystrophies that have been modeled using human retinal organoids include retinitis pigmentosa due to mutations in *RPGR* (Deng et al., 2018), *PRPF31* (Buskin et al., 2018), *USH2A* (Guo et al., 2019), and *RP2* (Lane et al., 2020).

Additionally, the combination of stem cell-derived RPE cultures with neural retinal organoids to recreate the native juxtaposition is an active field of research. Achberger et al. (2019) used an organ-on-a-chip technology to show that retinal organoid-RPE contact enhanced photoreceptor outer segment formation and re-established physiological processes including outer segment phagocytosis and calcium dynamics (Achberger et al., 2019). This could have important implications for modeling developmental, physiological, and disease processes that depend on the interaction between these tissues (Singh and Nasonkin, 2020).

Finally, retinal organoids have also been used to model retinoblastoma, the most prevalent intraocular malignancy in children, which has a developmental origin (Liu et al., 2020). Retinal organoids generated from hESC harboring biallelic mutations in the *RB1* gene developed tumor-like structures, and single-cell RNASeq analysis implicated ARR3-positive developing cone precursors as the cell of origin of these tumors. Additionally, the study found that inhibitors of spleen tyrosine kinase (SYK), which was significantly upregulated in this model, led to apoptosis in cancerous organoids, which could be relevant as a potential therapeutic agent (Liu et al., 2020).

Despite these and other encouraging results, the potential of organoids to study congenital retinal defects remains largely untapped. For instance, these models could be used to elucidate the mechanisms that lead to the retinal phenotype observed in conditions like Down syndrome, where progress has been slow due in part to the limitations of animal models in recapitulating human pathophysiology; and they could also contribute to our understanding of how viruses, toxins and other environmental exposures affect the human retina during embryonic development, as it has been described for human brain organoids. For example, infection with Zika virus, which causes fetal microcephaly, has been modeled in forebrain organoids from hiPSCs (Garcez et al., 2016; Qian et al., 2016). These studies showed preferential infection of neural progenitors which led to increased cell death and decreased proliferation, resulting in reduced neuronal cell-layer volume resembling microcephaly. Similar strategies could be used to establish the effect of the Zika virus in retinal organoids. Additionally, in the case of environmental toxins, Wang et al. (2018) used a brain organoid-on-a-chip system to simulate nervous system exposure to prenatal nicotine, and found that it can cause premature differentiation and apoptosis of neurons, with inhibition of neurite outgrowth and structural development of the cortex (Wang et al., 2018). Similar studies exploring the effect of environmental toxins in the retina are currently lacking.

## Conclusion

The cumulative knowledge of retina development has been instrumental in the generation of retinal organoid systems. The time is now ripe for retinal organoids to inform our understanding of retina development. This deeper understanding, combined with the advantages of retinal organoids as culture models that allow tight control of experimental manipulations and the possibility to model disease in a human setting, offer unique opportunities to gain insights into the pathophysiology of congenital retinal abnormalities for the development of potential therapeutic approaches.

## Author Contributions

MV and AV wrote the initial manuscript draft. YP and CS prepared the illustrations. All authors contributed to the article and approved the submitted version.

## Conflict of Interest

The authors declare that the research was conducted in the absence of any commercial or financial relationships that could be construed as a potential conflict of interest.

## References

[B1] Aasen D. M. Vergara M. N. (2020). New drug discovery paradigms for retinal diseases: a focus on retinal organoids. J. Ocul. Pharmacol. Ther. 36, 18–24. 10.1089/jop.2018.0140 31059378 PMC6985764

[B2] Achberger K. Probst C. Haderspeck J. Bolz S. Rogal J. Chuchuy J.. (2019). Merging organoid and organ-on-a-chip technology to generate complex multi-layer tissue models in a human retina-on-a-chip platform. eLife 8:e46188. 10.7554/eLife.46188 31451149 PMC6777939

[B3] Adler R. Canto-Soler M. V. (2007). Molecular mechanisms of optic vesicle development: complexities, ambiguities and controversies. Dev. Biol. 305, 1–13. 10.1016/j.ydbio.2007.01.045 17335797 PMC1927083

[B5] Brooks M. J. Chen H. Y. Kelley R. A. Mondal A. K. Nagashima K. De Val N.. (2019). Improved retinal organoid differentiation by modulating signaling pathways revealed by comparative transcriptome analyses with development *in vivo*. Stem Cell Rep. 13, 891–905. 10.1016/j.stemcr.2019.09.009 31631019 PMC6895716

[B6] Brzezinski J. A. Reh T. A. (2015). Photoreceptor cell fate specification in vertebrates. Development 142, 3263–3273. 10.1242/dev.127043 26443631 PMC4631758

[B8] Buskin A. Zhu L. Chichagova V. Basu B. Mozaffari-Jovin S. Dolan D.. (2018). Disrupted alternative splicing for genes implicated in splicing and ciliogenesis causes PRPF31 retinitis pigmentosa. Nat. Commun. 9:4234. 10.1038/s41467-018-06448-y 30315276 PMC6185938

[B9] Byerly M. S. Blackshaw S. (2009). Vertebrate retina and hypothalamus development. Wiley Interdiscip. Rev. Syst. Biol. Med. 1, 380–389. 10.1002/wsbm.22 20836003

[B10] Carpenter A. C. Smith A. N. Wagner H. Cohen-Tayar Y. Rao S. Wallace V.. (2015). Wnt ligands from the embryonic surface ectoderm regulate ‘bimetallic strip’ optic cup morphogenesis in mouse. Development 142, 972–982. 10.1242/dev.120022 25715397 PMC4352985

[B11] Cepko C. L. Austin C. P. Yang X. Alexiades M. Ezzeddine D. (1996). Cell fate determination in the vertebrate retina. Proc. Natl. Acad. Sci. U S A 93, 589–595. 10.1073/pnas.93.2.589 8570600 PMC40096

[B12] Collin J. Queen R. Zerti D. Dorgau B. Hussain R. Coxhead J.. (2019a). Deconstructing retinal organoids: single cell RNA-Seq reveals the cellular components of human pluripotent stem cell-derived retina. Stem Cells 37, 593–598. 10.1002/stem.2963 30548510 PMC6519347

[B13] Collin J. Zerti D. Queen R. Santos-Ferreira T. Bauer R. Coxhead J.. (2019b). CRX expression in pluripotent stem cell-derived photoreceptors marks a transplantable subpopulation of early cones. Stem Cells 37, 609–622. 10.1002/stem.2974 30681766 PMC6519156

[B14] Cowan C. S. Renner M. De Gennaro M. Gross-Scherf B. Goldblum D. Hou Y.. (2020). Cell types of the human retina and its organoids at single-cell resolution. Cell 182, 1623–1640.e1634. 10.1016/j.cell.2020.08.013 32946783 PMC7505495

[B15] Deng W. L. Gao M. L. Lei X. L. Lv J. N. Zhao H. He K. W.. (2018). Gene correction reverses ciliopathy and photoreceptor loss in iPSC-derived retinal organoids from retinitis pigmentosa patients. Stem Cell Rep. 10, 1267–1281. 10.1016/j.stemcr.2018.02.003 29526738 PMC5998840

[B16] Eiraku M. Takata N. Ishibashi H. Kawada M. Sakakura E. Okuda S.. (2011). Self-organizing optic-cup morphogenesis in three-dimensional culture. Nature 472, 51–56. 10.1038/nature09941 21475194

[B170] Eldred K. C. Hadyniak S. E. Hussey K. A. Brenerman B. Zhang P. W. Chamling X.. (2018). Thyroid hormone signaling specifies cone subtypes in human retinal organoids. Science 362:eaau6348. 10.1126/science.aau6348 30309916 PMC6249681

[B18] Fligor C. M. Langer K. B. Sridhar A. Ren Y. Shields P. K. Michael C. E.. (2018). Three-dimensional retinal organoids facilitate the investigation of retinal ganglion cell development, organization and neurite outgrowth from human pluripotent stem cells. Sci. Rep. 8:14520. 10.1038/s41598-018-32871-8 30266927 PMC6162218

[B19] Fuhrmann S. (2008). Wnt signaling in eye organogenesis. Organogenesis 4, 60–67. 10.4161/org.4.2.5850 19122781 PMC2613311

[B20] Fuhrmann S. (2010). Eye morphogenesis and patterning of the optic vesicle. Curr. Top. Dev. Biol. 93, 61–84. 10.1016/B978-0-12-385044-7.00003-5 20959163 PMC2958684

[B21] Fuhrmann S. Levine E. M. Reh T. A. (2000). Extraocular mesenchyme patterns the optic vesicle during early eye development in the embryonic chick. Development 127, 4599–4609. 10.1242/dev.127.21.4599 11023863

[B22] Garcez P. P. Loiola E. C. Madeiro Da Costa R. Higa L. M. Trindade P. Delvecchio R.. (2016). Zika virus impairs growth in human neurospheres and brain organoids. Science 352, 816–818. 10.1126/science.aaf6116 27064148

[B23] Guo Y. Wang P. Ma J. H. Cui Z. Yu Q. Liu S.. (2019). Modeling retinitis pigmentosa: retinal organoids generated from the iPSCs of a patient with the USH2A mutation show early developmental abnormalities. Front. Cell. Neurosci. 13:361. 10.3389/fncel.2019.00361 31481876 PMC6709881

[B24] Hallam D. Hilgen G. Dorgau B. Zhu L. Yu M. Bojic S.. (2018). Human-induced pluripotent stem cells generate light responsive retinal organoids with variable and nutrient-dependent efficiency. Stem Cells 36, 1535–1551. 10.1002/stem.2883 30004612 PMC6392112

[B25] Heavner W. Pevny L. (2012). Eye development and retinogenesis. Cold. Spring. Harb. Perspect. Biol. 4:a008391. 10.1101/cshperspect.a008391 23071378 PMC3504437

[B27] Horsford D. J. Nguyen M. T. Sellar G. C. Kothary R. Arnheiter H. Mcinnes R. R.. (2005). Chx10 repression of Mitf is required for the maintenance of mammalian neuroretinal identity. Development 132, 177–187. 10.1242/dev.01571 15576400

[B28] Hoshino A. Ratnapriya R. Brooks M. J. Chaitankar V. Wilken M. S. Zhang C.. (2017). Molecular anatomy of the developing human retina. Dev. Cell 43, 763–779.e764. 10.1016/j.devcel.2017.10.029 29233477 PMC5776731

[B29] Hyer J. Mima T. Mikawa T. (1998). FGF1 patterns the optic vesicle by directing the placement of the neural retina domain. Development 125, 869–877. 10.1242/dev.125.5.869 9449669

[B30] Kagiyama Y. Gotouda N. Sakagami K. Yasuda K. Mochii M. Araki M.. (2005). Extraocular dorsal signal affects the developmental fate of the optic vesicle and patterns the optic neuroepithelium. Dev. Growth Differ. 47, 523–536. 10.1111/j.1440-169X.2005.00828.x 16287484

[B31] Kallman A. Capowski E. E. Wang J. Kaushik A. M. Jansen A. D. Edwards K. L.. (2020). Investigating cone photoreceptor development using patient-derived NRL null retinal organoids. Commun. Biol. 3:82. 10.1038/s42003-020-0808-5 32081919 PMC7035245

[B32] Kaya K. D. Chen H. Y. Brooks M. J. Kelley R. A. Shimada H. Nagashima K.. (2019). Transcriptome-based molecular staging of human stem cell-derived retinal organoids uncovers accelerated photoreceptor differentiation by 9-cis retinal. Mol. Vis. 25, 663–678. Available online at: http://www.molvis.org/molvis/v25/663. 31814692 PMC6857775

[B34] Kim S. Lowe A. Dharmat R. Lee S. Owen L. A. Wang J.. (2019). Generation, transcriptome profiling and functional validation of cone-rich human retinal organoids. Proc. Natl. Acad. Sci. U S A 116, 10824–10833. 10.1073/pnas.1901572116 31072937 PMC6561190

[B35] Lane A. Jovanovic K. Shortall C. Ottaviani D. Panes A. B. Schwarz N.. (2020). Modeling and rescue of RP2 retinitis pigmentosa using iPSC-derived retinal organoids. Stem Cell Rep. 15, 67–79. 10.1016/j.stemcr.2020.05.007 32531192 PMC7363745

[B36] Langer K. B. Ohlemacher S. K. Phillips M. J. Fligor C. M. Jiang P. Gamm D. M.. (2018). Retinal ganglion cell diversity and subtype specification from human pluripotent stem cells. Stem Cell Rep. 10, 1282–1293. 10.1016/j.stemcr.2018.02.010 29576537 PMC5998302

[B37] Littink K. W. Stappers P. T. Y. Riemslag F. C. C. Talsma H. E. Van Genderen M. M. Cremers F. P. M.. (2018). Autosomal recessive nrl mutations in patients with enhanced s-cone syndrome. Genes 9:68. 10.3390/genes9020068 29385733 PMC5852564

[B38] Liu H. Zhang Y. Zhang Y. Y. Li Y. P. Hua Z. Q. Zhang C. J.. (2020). Human embryonic stem cell-derived organoid retinoblastoma reveals a cancerous origin. Proc. Natl. Acad. Sci. U S A 117, 33628–33638. 10.1073/pnas.2011780117 33318192 PMC7776986

[B39] Luo Z. Xu C. Li K. Xian B. Liu Y. Li K.. (2019). Islet1 and Brn3 expression pattern study in human retina and hiPSC-derived retinal organoid. Stem Cells Int. 2019:8786396. 10.1155/2019/8786396 31885629 PMC6925930

[B43] Mellough C. B. Collin J. Queen R. Hilgen G. Dorgau B. Zerti D.. (2019). Systematic comparison of retinal organoid differentiation from human pluripotent stem cells reveals stage specific, cell line and methodological differences. Stem Cells Transl. Med. 8, 694–706. 10.1002/sctm.18-0267 30916455 PMC6591558

[B44] Meyer J. S. Shearer R. L. Capowski E. E. Wright L. S. Wallace K. A. Mcmillan E. L.. (2009). Modeling early retinal development with human embryonic and induced pluripotent stem cells. Proc. Natl. Acad. Sci. U S A 106, 16698–16703. 10.1073/pnas.0905245106 19706890 PMC2757802

[B46] Nakano T. Ando S. Takata N. Kawada M. Muguruma K. Sekiguchi K.. (2012). Self-formation of optic cups and storable stratified neural retina from human ESCs. Cell Stem Cell 10, 771–785. 10.1016/j.stem.2012.05.009 22704518

[B47] Nguyen M. Arnheiter H. (2000). Signaling and transcriptional regulation in early mammalian eye development: a link between FGF and MITF. Development 127, 3581–3591. 10.1242/dev.127.16.3581 10903182

[B48] Nishiguchi K. M. Friedman J. S. Sandberg M. A. Swaroop A. Berson E. L. Dryja T. P.. (2004). Recessive NRL mutations in patients with clumped pigmentary retinal degeneration and relative preservation of blue cone function. Proc. Natl. Acad. Sci. U S A 101, 17819–17824. 10.1073/pnas.0408183101 15591106 PMC535407

[B49] O’hara-Wright M. Gonzalez-Cordero A. (2020). Retinal organoids: a window into human retinal development. Development 147:dev189746. 10.1242/dev.189746 33361444 PMC7774906

[B50] Ohlemacher S. K. Sridhar A. Xiao Y. Hochstetler A. E. Sarfarazi M. Cummins T. R.. (2016). Stepwise differentiation of retinal ganglion cells from human pluripotent stem cells enables analysis of glaucomatous neurodegeneration. Stem Cells 34, 1553–1562. 10.1002/stem.2356 26996528 PMC4892962

[B51] Parfitt D. A. Lane A. Ramsden C. M. Carr A. F. Munro P. M. Jovanovic K.. (2016). Identification and correction of mechanisms underlying inherited blindness in human iPSC-derived optic cups. Cell Stem Cell 18, 769–781. 10.1016/j.stem.2016.03.021 27151457 PMC4899423

[B52] Phillips M. J. Perez E. T. Martin J. M. Reshel S. T. Wallace K. A. Capowski E. E.. (2014). Modeling human retinal development with patient-specific induced pluripotent stem cells reveals multiple roles for visual system homeobox 2. Stem Cells 32, 1480–1492. 10.1002/stem.1667 24532057 PMC4037340

[B53] Pittack C. Grunwald G. B. Reh T. A. (1997). Fibroblast growth factors are necessary for neural retina but not pigmented epithelium differentiation in chick embryos. Development 124, 805–816. 10.1242/dev.124.4.805 9043062

[B54] Qian X. Nguyen H. N. Song M. M. Hadiono C. Ogden S. C. Hammack C.. (2016). Brain-region-specific organoids using mini-bioreactors for modeling ZIKV exposure. Cell 165, 1238–1254. 10.1016/j.cell.2016.04.032 27118425 PMC4900885

[B55] Rachel R. A. Yamamoto E. A. Dewanjee M. K. May-Simera H. L. Sergeev Y. V. Hackett A. N.. (2015). CEP290 alleles in mice disrupt tissue-specific cilia biogenesis and recapitulate features of syndromic ciliopathies. Hum. Mol. Genet. 24, 3775–3791. 10.1093/hmg/ddv123 25859007 PMC4459394

[B56] Shimada H. Lu Q. Insinna-Kettenhofen C. Nagashima K. English M. A. Semler E. M.. (2017). *in vitro* modeling using ciliopathy-patient-derived cells reveals distinct cilia dysfunctions caused by CEP290 mutations. Cell Rep. 20, 384–396. 10.1016/j.celrep.2017.06.045 28700940 PMC5553702

[B57] Singh R. K. Mallela R. K. Cornuet P. K. Reifler A. N. Chervenak A. P. West M. D.. (2015). Characterization of three-dimensional retinal tissue derived from human embryonic stem cells in adherent monolayer cultures. Stem Cells Dev. 24, 2778–2795. 10.1089/scd.2015.0144 26283078 PMC4653822

[B171] Singh R. K. Nasonkin I. O. (2020). Limitations and promise of retinal tissue from human pluripotent stem cells for developing therapies of blindness. Front. Cell. Neurosci. 14:179. 10.3389/fncel.2020.00179 33132839 PMC7513806

[B58] Singh R. K. Occelli L. M. Binette F. Petersen-Jones S. M. Nasonkin I. O. (2019). Transplantation of human embryonic stem cell-derived retinal tissue in the subretinal space of the cat eye. Stem Cells Dev. 28, 1151–1166. 10.1089/scd.2019.0090 31210100 PMC6708274

[B59] Steinfeld J. Steinfeld I. Coronato N. Hampel M. L. Layer P. G. Araki M.. (2013). RPE specification in the chick is mediated by surface ectoderm-derived BMP and Wnt signalling. Development 140, 4959–4969. 10.1242/dev.096990 24227655

[B62] Takata N. Abbey D. Fiore L. Acosta S. Feng R. Gil H. J.. (2017). An eye organoid approach identifies Six3 suppression of R-spondin 2 as a critical step in mouse neuroretina differentiation. Cell Rep. 21, 1534–1549. 10.1016/j.celrep.2017.10.041 29117559 PMC5728169

[B63] VanderWall K. B. Huang K. C. Pan Y. Lavekar S. S. Fligor C. M. Allsop A. R.. (2020). Retinal ganglion cells with a glaucoma OPTN(E50K) mutation exhibit neurodegenerative phenotypes when derived from three-dimensional retinal organoids. Stem Cell Rep. 15, 52–66. 10.1016/j.stemcr.2020.05.009 32531194 PMC7363877

[B64] Vergara M. N. Flores-Bellver M. Aparicio-Domingo S. Mcnally M. Wahlin K. J. Saxena M. T.. (2017). Three-dimensional automated reporter quantification (3D-ARQ) technology enables quantitative screening in retinal organoids. Development 144, 3698–3705. 10.1242/dev.146290 28870990 PMC5675442

[B65] Volkner M. Zschatzsch M. Rostovskaya M. Overall R. W. Busskamp V. Anastassiadis K.. (2016). Retinal organoids from pluripotent stem cells efficiently recapitulate retinogenesis. Stem Cell Rep. 6, 525–538. 10.1016/j.stemcr.2016.03.001 27050948 PMC4834051

[B66] Wahlin K. J. Maruotti J. A. Sripathi S. R. Ball J. Angueyra J. M. Kim C.. (2017). Photoreceptor outer segment-like structures in long-term 3D retinas from human pluripotent stem cells. Sci. Rep. 7:766. 10.1038/s41598-017-00774-9 28396597 PMC5429674

[B67] Wang Y. Wang L. Zhu Y. Qin J. (2018). Human brain organoid-on-a-chip to model prenatal nicotine exposure. Lab Chip 18, 851–860. 10.1039/c7lc01084b 29437173

[B68] Westenskow P. Piccolo S. Fuhrmann S. (2009). Beta-catenin controls differentiation of the retinal pigment epithelium in the mouse optic cup by regulating Mitf and Otx2 expression. Development 136, 2505–2510. 10.1242/dev.032136 19553286 PMC2709060

[B69] Zhong X. Gutierrez C. Xue T. Hampton C. Vergara M. N. Cao L. H.. (2014). Generation of three-dimensional retinal tissue with functional photoreceptors from human iPSCs. Nat. Commun. 5:4047. 10.1038/ncomms5047 24915161 PMC4370190

[B70] Zuber M. E. Gestri G. Viczian A. S. Barsacchi G. Harris W. A. (2003). Specification of the vertebrate eye by a network of eye field transcription factors. Development 130, 5155–5167. 10.1242/dev.00723 12944429

